# Emprint Microwave Thermoablation System: Bridging Thermal Ablation Efficacy between Human Patients and Porcine Models through Mathematical Correlation

**DOI:** 10.3390/bioengineering10091057

**Published:** 2023-09-07

**Authors:** Andrea Cafarchio, Marcello Iasiello, Maria Chiara Brunese, Giampiero Francica, Aldo Rocca, Assunta Andreozzi

**Affiliations:** 1 Dipartimento di Medicina e Scienze della Salute DIMES, Università degli Studi del Molise, 86100 Campobasso, Italy; mariachiarabrunese@gmail.com (M.C.B.); aldorocca@unimol.it (A.R.); 2 Dipartimento di Ingegneria Industriale DII, Università degli Studi di Napoli “Federico II”, 80125 Napoli, Italy; marcello.iasiello@unina.it (M.I.); asandreo@unina.it (A.A.); 3 Interventional Ultrasound Unit, Pineta Grande Hospital, 81030 Castel Volturno, Italy; giampierofrancica@gmail.com

**Keywords:** microwave ablation, hyperthermia, blood perfusion, Emprint, ablation volume

## Abstract

To investigate the in vivo ablation characteristics of a microwave ablation antenna in the livers of humans with tumors, a retrospective analysis of the ablation zones was conducted after applying Emprint microwave ablation systems for treatment. Percutaneous microwave ablations performed between January 2022 and September 2022 were included in this study. Subsequently, immediate post-ablation echography images were subjected to retrospective evaluation to state the long ablated diameter, short ablated diameter, and volume. The calculated ablation lengths and volume indices were then compared between in vivo and ex vivo results obtained from laboratory experiments conducted on porcine liver. The ex vivo data showed a good correlation between energy delivered and both increasing ablated dimensions (both *p* < 0.001) and volume (*p* < 0.001). The in vivo data showed a good correlation for dimensions (*p* = 0.037 and *p* = 0.019) and a worse correlation for volume (*p* = 0.142). When comparing ex vivo and in vivo data for higher energies, the ablated volumes grew much more rapidly in ex vivo cases compared to in vivo ones. Finally, a set of correlations to scale ex vivo results with in vivo ones is presented. This phenomenon was likely due to the absence of perfusion, which acts as a cooling system.

## 1. Introduction

Thermal ablation has emerged as a widely accepted treatment modality for liver malignancies, offering a less invasive alternative to surgical resection for small lesions in both primary and secondary liver tumors [1,2]. While radiofrequency ablation (RFA) has traditionally been the predominant technique, microwave ablation (MWA) has gained significant popularity in recent years [3,4]. RFA and MWA are minimally invasive therapeutic techniques that employ thermal energy to treat various medical conditions, such as solid tumors. These techniques aim to induce coagulative necrosis within the targeted tissue by generating localized heat. Despite sharing this common objective, RFA and MWA differ in their mechanisms of heat generation, influencing their clinical applications and outcomes. RFA operates by utilizing an alternating current to generate heat within tissues. An electrode is inserted into the target tissue, and radiofrequency energy is applied through the electrode, leading to resistive heating. The oscillating electromagnetic field causes ions within the tissue to rotate rapidly, resulting in friction and subsequent heat production. The heat generated is conducted through the tissue, causing protein denaturation and cell death. RFA typically operates at lower frequencies (approximately 400 kHz), limiting the depth of energy penetration and potentially leading to heat sink effects near large blood vessels. MWA, on the other hand, employs electromagnetic waves at microwave frequencies (typically 900 MHz to 2.45 GHz) to generate heat. This technique utilizes an antenna to emit microwaves, which cause water molecules in the tissue to oscillate and generate heat through dipolar rotation. Unlike RFA, MWA generates heat directly within the tissue’s water molecules, leading to rapid and more volumetric heating. The unique properties of MWA result in several advantages over RFA. MWA achieves higher temperatures, enabling larger ablation zones in shorter durations while demonstrating reduced susceptibility to the surrounding tissue properties [4,5]. Additionally, MWA encounters fewer challenges when propagating through (cirrhotic) tissue with high impedance and during ablations near intrahepatic vessels, minimizing heat-sink effects [5,6]. Furthermore, MWA eliminates the need for grounding pads, mitigating the risk of skin burns [7].

Nonetheless, MWA does come with certain disadvantages. In comparison to RFA, the shape of MWA ablation zones has been reported to be more elliptical rather than spherical [8]. Additionally, the size and shape of coagulation necrosis produced by MWA tend to be less predictable [8]. Achieving predictability is crucial for attaining favorable outcomes. However, with the utilization of advanced planning and navigation tools, oncological outcomes comparable to surgical resection can be achieved [9,10]. Interventional radiologists now have access to highly sophisticated navigation software and robotic assistance, enabling optimized planning and precise needle placement guidance [11,12]. These tools utilize modeling techniques that rely on the prerequisite of predictability in terms of ablation shape and volume. Ablation systems are equipped with predefined algorithms to estimate the size and shape of the ablation, and manufacturers provide reference values for ablations at different settings. However, in practice, these theoretical reference values often deviate from the actual dimensions of the coagulated tissue [13].

Numerous studies are dedicated to the development of predictive models employing mathematical equations to effectively approximate complex problems. For example, in research conducted by Andreozzi et al. in 2019 [14], an evaluation was performed to compare various mathematical models routinely employed in thermoablation. The obtained outcomes, derived from the implementation of Penne’s equation, local thermal non-equilibrium (LTNE) equations, and a three-phase model, were compared with experimental data. Moreover, in 2021, Tucci et al. [15] conducted a similar comparison, introducing a porous model for the analyzed tissue in an attempt to improve the performance of the mathematical models. In 2021, Singh et al. [16] conducted a modeling study aiming to optimize the geometry of the simulated domain to achieve results that closely match real-world conditions. Tucci et al. [17] presented a novel model in 2022, where they introduced spatially varying porosity within the tissues to provide a more accurate simulation of the tumor microenvironment. Winokour et al. [18] conducted a study to investigate the reliability of manufacturer-provided data for predictive purposes during surgery. The results showed that the provided measurements by the manufacturer are not suitable for accurately predicting the antenna’s behavior in vivo during the preoperative phase.

Considering the observed disparities between in vivo and in vitro values as documented in the existing body of literature, the primary objective of this research endeavor is to delve into the fundamental factors contributing to these incongruities. Notably, it is worth emphasizing that among these factors, perfusion emerges as a prominent determinant. Concurrently, the study aims to proffer a mathematical model conducive to the calibration of ex vivo datasets. This calibration is intended to facilitate their extrapolation for the purpose of anticipatory projection regarding the shape and dimensions of the ablated region during in vivo patient surgery. Specifically, the Emprint Ablation System with an Emprint Ablation Antenna [19] was employed in both ex vivo and in vivo experiments to analyze the role of blood perfusion on the size and shape of the ablation zone. Emprint is a system that utilizes microwave technology to transmit energy through a soft tissue-penetrating antenna. Its primary function is to coagulate (ablate) a specific volume of tissue. The Emprint Ablation System employs a generator to supply power to a single microwave ablation antenna. By incorporating the patented Thermosphere [19] technology, it ensures a consistent ablation zone concentrated at the antenna’s tip, regardless of varying tissue conditions. This technology is beneficial in reducing the likelihood of local tumor recurrence by achieving large ablation volumes with reliable margins. To evaluate the role of perfusion on the Emprint ablation system, a controlled ex vivo study using non-perfused, healthy porcine livers with standardized needle placement was conducted. The use of an ex vivo study protocol allowed to isolate the impact of factors unrelated to the design and technology of the MWA systems. For comparison purposes on perfusion role during ablation treatments, human in vivo data provided by Clinica Pineta Grande Castel Volturno CE, Italy were employed. This experimental study aimed to investigate how the perfusion affects ex vivo and in vivo data in terms of sphericity and ablation size. By analyzing these parameters, the paper aim was to gain insights into the respective capabilities and limitations of the EMPRINT systems in achieving desirable ablation outcomes. The Emprint antenna was utilized in this study, but the methodology can be extended to other models given enough ex vivo and in vivo data.

## 2. Materials and Methods

### 2.1. Microwave Ablation Systems

The employed system was the Emprint Ablation System, featuring a generator capable of delivering a maximum power output of 100 W at a frequency of 2.45 GHz. The system incorporated a perfusion cooled antenna and a flexible coaxial cable. The Emprint system was combined with an 11-gauge Emprint antenna with a length of 150 mm. It is important to note that the manufacturer had no involvement in the execution or analysis of this study. The same ablation system was used for both ex vivo and in vivo experiments.

### 2.2. Ex Vivo Ablation Protocol

**Figure 1:**
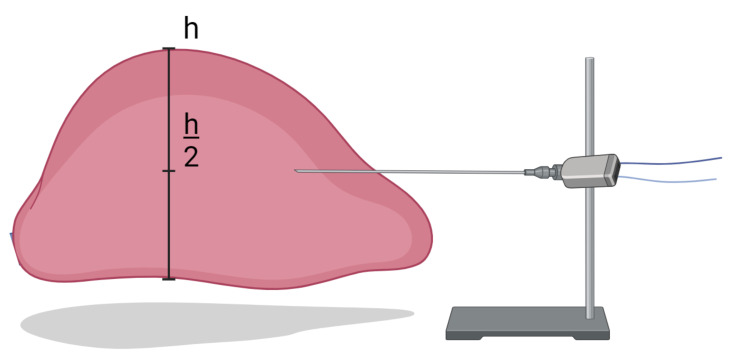
A sketch experimental ablation setup. The liver lobe was placed inside a plastic container. The antenna was inserted at half the height of the liver in the horizontal position, fixed with a support.

In the ex vivo animal investigation, a total of 11 porcine livers were investigated preserved in a 0.9% NaCl solution at a temperature of 4 °C. Each porcine liver was divided into four parts, representing the four largest porcine liver lobes (left/right medial and lateral lobes). In each experiment, the region of interest had to exceed the expected ablated area by at least 5 mm on all sides. Each liver lobe was positioned and fixed for an upright position, as shown in the schematic representation in Figure 1. An insertion point for the horizontal microwave ablation (MWA) antenna was selected at the midpoint of both the height and width of the liver lobe. The antenna was inserted with a minimum depth of 50 mm. This antenna placement setup was used to ensure sufficient tissue coverage in all the directions from the antenna slot, thereby preventing heat dissipation outside the tissue. Figure 1 also illustrates the placement of the antenna, ensuring stability by firmly securing the handlebar during the ablation process.

**Figure 2:**
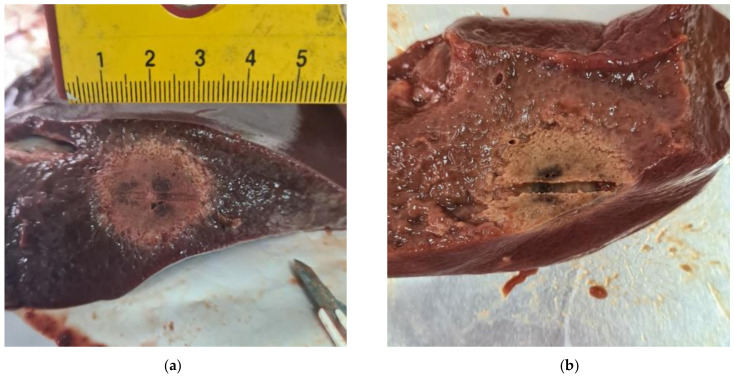
Results of ex vivo experiments. (**a**) The ablated area does not touch the tissue surface, with a margin of at least 5 mm on each side; this result was used within the present analysis. (**b**) The ablated area touches the tissue surface; this result was not used for the present analysis.

Ablations were performed at 9 different settings: ranging between 2 and 6 min of ablation time and between 160 and 80 watts. An ablation was considered eligible for analysis if it successfully completed the intended ablation time and did not extend to the surface of the liver (Figure 2).

It is important to exclude experiments that involve direct contact with the tissue surface because when this occurs, there is non-isotropic heat transmission. This is due to the changing boundary conditions caused by the different heat transmission properties between the external air and the tissue. A total of 44 ablations were performed, of which 15 were excluded due to reaching the liver surface, and 2 for a failure in the cooling system. Finally, 27 successful ablations were available for analysis: 3 for each setting.

### 2.3. In Vivo Ablation Protocol

Between January 2022 and September 2022, a prospective enrollment of 22 patients diagnosed with a solid liver lesion was conducted at Pineta Grande Hospital, Castel Volturno, CE. Out of the 22 enrolled patients, 9 individuals underwent treatment for liver metastases originating from colorectal cancer, while the remaining 13 patients received treatment for hepatocellular carcinoma (HCC). Only the data from patients diagnosed with HCC were utilized for statistical analyses because of the similarity in properties with the employed ex vivo tissue. The microwave ablation treatment was exclusively employed for patients whose lesions had a maximum diameter of 3 cm. The protocol for microwave thermal ablation with an antenna involved the following steps: Prior to the procedure, informed consent was obtained from each patient after a thorough assessment of their medical history and imaging evaluation to ensure suitability for microwave thermal ablation.

**Figure 3:**
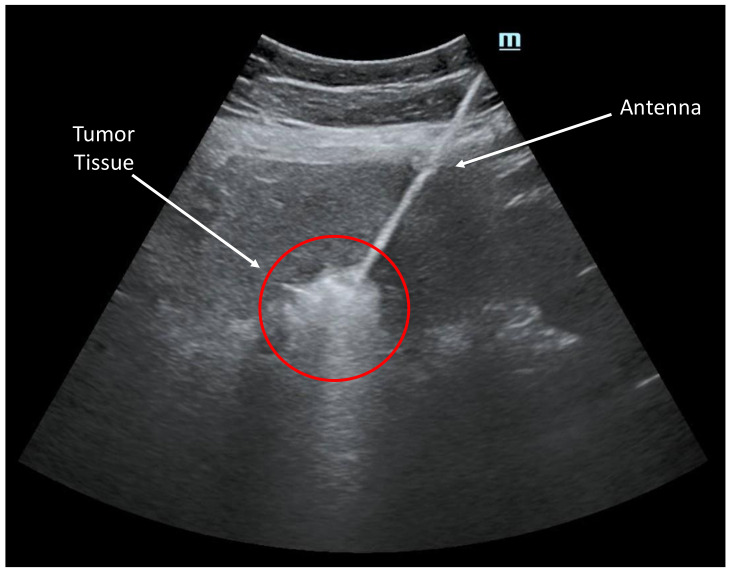
Ultrasound image showcasing an ablation procedure in progress. The image captures the targeted area where a catheter was guided to deliver precise energy to remove abnormal tissue. Real-time visualization provided by ultrasound technology allowed for accurate placement of the catheter, ensuring optimal treatment efficacy.

The Emprint microwave ablation system and Emprint antenna were selected based on the characteristics of the target lesion. The system was prepared, ensuring proper functioning and calibration. Power and treatment time parameters were set based on the size and location of the lesion. Anesthesia was administered according to the patient’s needs and the physician’s discretion, which could involve local anesthesia or conscious sedation. Under ultrasound image guidance (Figure 3), the antenna was accurately positioned within the target lesion. The optimal insertion point and trajectory were determined, followed by a percutaneous insertion of the antenna using a minimally invasive approach, guided by real-time imaging. Once the antenna was in place, the microwave ablation system was activated to deliver the predetermined power and treatment time. The ablation process was carefully monitored to ensure proper tissue heating within the targeted region. If necessary, adjustments were made to the antenna position to achieve complete coverage of the lesion.

### 2.4. Assessment of Ablation Size and Geometry

To obtain volumetric data from the ablation necrosis in ex vivo experiments, the middle of the ablated volume was cut for all ablated liver lobes using a surgeon’s scalpel. To better observe the antenna slot’s effect and assess its final position within the tissue, the incision was made out along the direction of antenna insertion. Image processing was carried out with Fiji software to evaluate the size and shape of each ablation. The ablated volume was measured in cubic millimeters using a distance measuring tool, with the scale set based on a ruler placed near the sample.

Regarding the in vivo experiments, volumetric data on the ablation necrosis were calculated from the ultrasound images. The ultrasound, despite computer tomography (CT) or magnetic resonance imaging (MRI), allows for a real-time evaluation of the lesion during the surgery and a precise post-operative dimensions evaluation. In addition, it was used as contrast-enhanced ultrasound (CEUS) that allowed for an even better evaluation of lesion margins. Furthermore, ultrasound is more cost-effective compared to CT and MRI, which incur high costs for public healthcare, and it exerts a lesser impact on the patient. This impact is both psychological (i.e., comfort) and in terms of the amount of radiation absorbed by the patient. In the context of the performed lesion ablation, it was important to consider the potential artifacts that could arise under ultrasound imaging post-ablation. Lesion-induced artifacts are common and can sometimes lead to challenges in accurately assessing the extent of the ablated tissue. To confirm the ablation range and enhance the reliability of our evaluation, we employed the following confirmation criteria during ultrasound imaging: A hypoechoic zone surrounding the ablation site was identified as an immediate indication of tissue alteration due to the ablation process. This zone typically exhibits decreased echogenicity compared to that of the surrounding tissues. Careful attention was paid to the margins of the ablated area. Clear demarcation between the treated region and the neighboring tissues suggested successful targeting of the lesion. Ultrasound imaging was performed in real time during the ablation procedure to monitor the progression and extent of tissue alteration. This allowed for immediate adjustments if needed. Multiple imaging planes, including longitudinal, transverse, and oblique views, were utilized to accurately visualize the ablation site from different angles. This comprehensive approach reduced the likelihood of overlooking any residual or incompletely treated areas. By adhering to these confirmation criteria, we aimed to mitigate the potential impact of artifacts and enhance the accuracy of our assessment regarding the ablation range.

**Figure 4:**
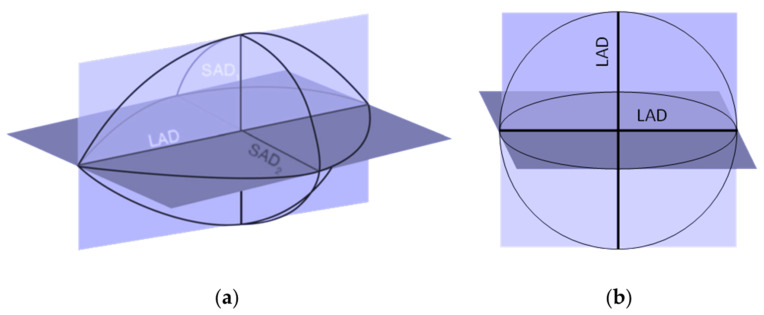
(**a**) Long ablated diameter (LAD) and short ablated diameter (SAD). (**b**) To calculate the sphericity index, an ideal sphere of diameter equal to the LAD was considered.

The diameter of the ablation was measured in three axes, as depicted in Figure 4: a long-axis diameter (LAD) aligned with the needle insertion axis, and two orthogonal short-axis diameters (SAD). The sphericity index (SI) was determined as the ratio between these diameters SAD/LAD. An SI value of 1 indicates a perfectly spherical ablation, while a lower or higher SI suggests a more elliptical shape of the ablation. The ablation volume (AV) was calculated using the ellipsoid volume formula V = 4/3∙π∙LAD∙[SAD]^2^, valid if two axes are equal, as stated by Hines-Peralta et al. [20] and Bartoletti et al. [21].

### 2.5. Ex Vivo vs. In Vivo Physical Properties

The thermal and electrical properties of liver tissue play a crucial role in thermal ablation therapies. Indeed, it has been shown that a correct experimentation for these studies requires the right replacement of properties, with particular references to the electrical ones [21]. Studies have shown that human liver tissue has a thermal conductivity ranging from approximately 0.4 to 0.6 W/m·K. On the other hand, porcine liver tissue demonstrates a slightly higher range of thermal conductivity, typically between 0.5 and 0.7 W/m·K between 22 and 82 °C [22,23]. Similarly, electrical conductivity, which indicates how well tissue conducts an electrical current, also differs between human and porcine liver. Human liver tissue exhibits an electrical conductivity ranging from approximately 0.33 to 0.59 S/m. In contrast, pig liver tissue demonstrates a slightly lower electrical conductivity, typically between 0.25 and 0.50 S/m [22,24]. Despite the use of porcine liver as a model or approximation for human liver being possible due to their similarities, it should be approached with caution, and the results should be interpreted and extrapolated with careful consideration of the limitations and potential discrepancies between them.

### 2.6. Statistical Analysis

The energy required for ablation was correlated with the ablation zone dimensions using Spearman’s rank correlation coefficient (r) [25]. Spearman’s rank correlation coefficient is a statistical measure used to assess the degree of association between two ordinal variables. In other words, it is employed to determine how two sets of ranked data are consistent with each other. This correlation coefficient is based on the ranks of observations and can be useful when data does not meet the assumptions of normality or when relationships between variables are non-linear. The median and interquartile range were used to represent continuous variables. Two-tailed Mann–Whitney U tests were employed to compare the distributional differences of continuous variables. For each statistical test, the *p*-value associated with the observed result was calculated. If the *p*-value is low, then it is considered that the data provide sufficient evidence to reject the null hypothesis and accept the alternative hypothesis. In other words, a low *p*-value indicates that the result is statistically significant and that the observed effect or difference is likely not due to chance but is probably real and replicable. In this study, a significance level of the *p*-value equal to 0.05 was considered to be statistically significant. The statistical analysis was performed using R software.

## 3. Results

In this section, the results obtained from the experiments are presented in terms of characteristic lesion dimensions (LAD, SAD, AV) as a function of treatment energy. Spearman’s rank correlation coefficients (r) between energy used and LAD, SAD, and AV were found to be 0.963 (*p* < 0.001), 0.974 (*p* < 0.001), and 0.977 (*p* < 0.001), respectively, showing a good correlation between energy delivered and lesion dimensions. Regarding in vivo ablation measurements, there were moderate but statistically significant correlations between increasing energy of ablation and increasing LAD (r = 0.688, *p* = 0.019) and SAD (r = 0.630, *p* = 0.037); on the other hand, the rank correlation was a little bit lower for volume (r = 0.498, *p* = 0.142).

The results presented in this section, both ex vivo and in vivo, were collected for energy values in the 0–24 kJ range. Table 1 displays the data obtained from ex vivo and in vivo experiments. The division into three energy ranges was made to facilitate a more direct comparison of data across different ranges of increasing energy and to appreciate the differences in values obtained when the treatment energy changed. These three ranges were selected to ensure the same data density in both ex vivo and in vivo conditions.

**Figure 5:**
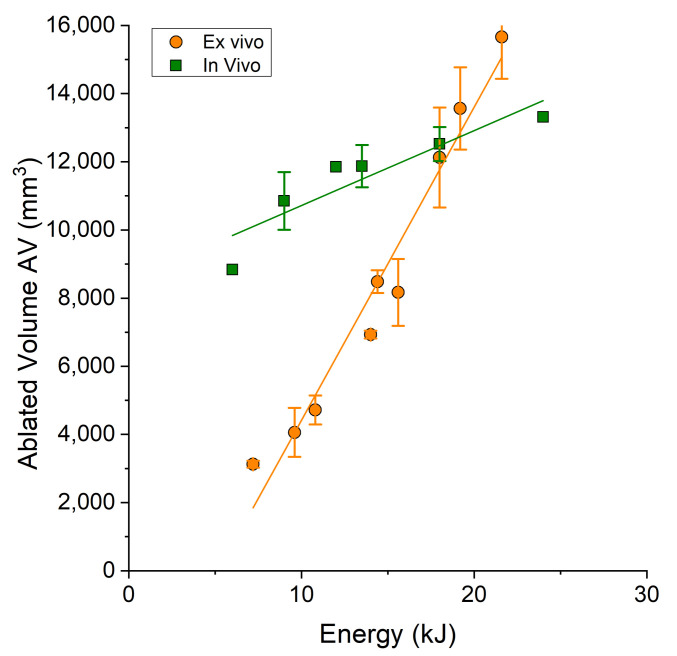
The volume of the ablated area as a function of the energy applied to the treatment. The orange data points represent the results from ex vivo experiments, while the green data points represent the results from in vivo experiments.

It is evident that the average size of the ablated areas in ex vivo tissue was smaller compared to that in in vivo tissue in the first two ranges. However, in the third range, corresponding to energy levels greater than 18 kJ, there was a reversal of this trend. Figure 5 illustrates the trend for energy values ranging from 0 to 24 kJ.

Table 2 and Table 3 show data about the LAD and SAD for ex vivo and in vivo ablation. Similarly, to ablation volume, there was a specific energy value at which a reversal of the trend occurred for both the LAD and SAD. This value was 17 kJ for LAD and 19 kJ for SAD, respectively.

**Figure 6:**
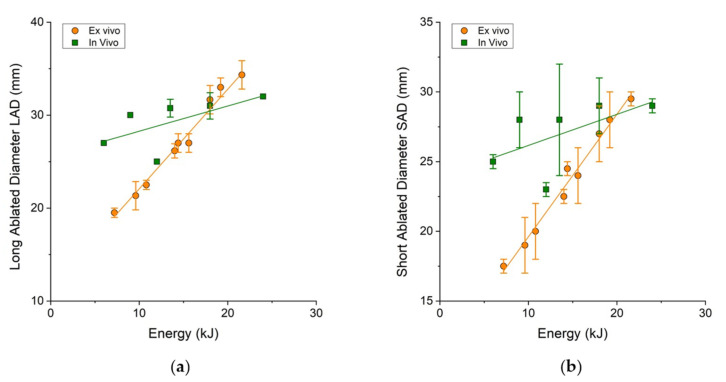
(**a**) The long ablated diameter (LAD) and (**b**) short ablated diameter (SAD) as a function of the energy applied to the treatment. The orange data points represent the results from ex vivo experiments, while the green data points represent the results from in vivo experiments.

**Figure 7:**
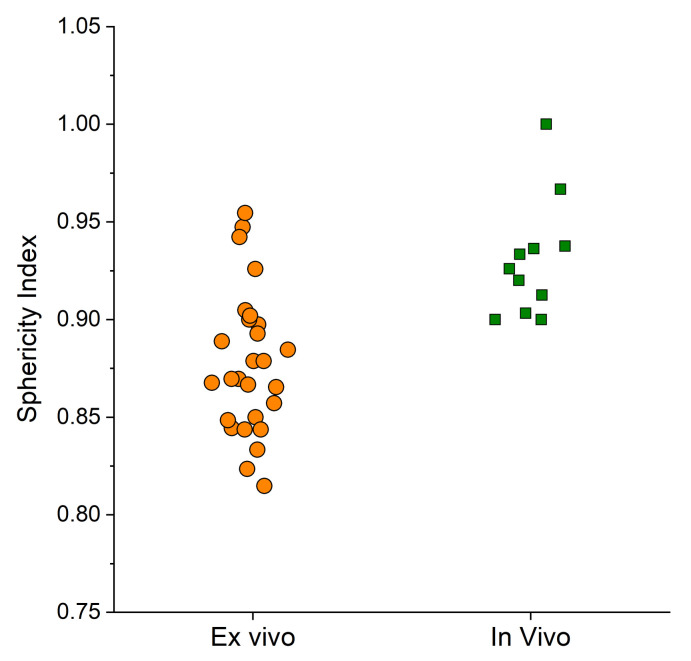
Sphericity index values for ex vivo and in vivo experiments.

The data for both LAD and SAD are shown in Figure 6. Finally, the sphericity index was calculated. Figure 7 shows that the values of the SI were similar in both ex vivo and in vivo data.

**Figure 8:**
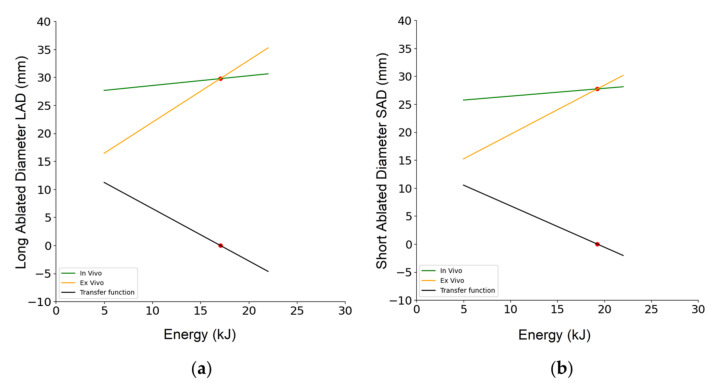
Transfer functions of LAD (**a**) and SAD (**b**) in the study. The plots show the relationship between the input variable and the predicted output variable. Notably, a red point was included in each graph, representing the inflection point where the trend reversed. In the case of LAD, the inflection point occurred at 17.014 kJ, while for SAD, it was observed at 17.022 kJ.

Despite the similarity, Spearman’s rank correlation coefficients between energy and the SI were found to be 0.419 (*p* < 0.029) for ex vivo data, suggesting that the association between the two variables would be considered statistically significant. On the other hand, in vivo data showed an r of 0.028 (*p* = 0.934), so the correlation between energy and the SI would not be considered statistically significant. Finally, a transfer function was computed to convert ex vivo data to in vivo data, which could be used as a predictive model to account for deviations between ex vivo and in vivo data. This tool might be helpful to scale ex vivo data to reach realistic in vivo conditions by just tuning the perfusion, which was found to be the main reason of deviation among the experiments. First, regression lines were calculated to approximate the ex vivo and in vivo data. The regression lines approximating the ex vivo data had equations y = 1.107x + 10.905 with R^2^ = 0.95 and y = 0.8789x + 10.821 with R^2^ = 0.97 for LAD and SAD as the dependent variable y, respectively, with the energy delivered as the input variable x. Similarly, for the in vivo data, the regression lines were y = 0.1744x + 26.795 with R^2^ = 0.91 and y = 0.1403x + 25.041 with R^2^ = 0.89 for LAD and SAD, respectively. Therefore, the following relationships were derived to establish differences from ex vivo and in vivo regressed data for LAD and SAD:

(1) $$\left\{\begin{array}{c} \mathrm{LAD}=0.9326\cdot E-15.89\\ \mathrm{SAD}=0.7386\cdot E-14.22 \end{array}\right.$$

with E as the energy to be delivered (the product between input power and time) and LAD and SAD in millimeters. These relationships can be used to make predictions about the ablated area in patients in vivo if only ex vivo data collected in the laboratory are available. Figure 8 shows the transfer functions for LAD (Figure 8a) and SAD (Figure 8b). Of course, negative points underline the fact that in some cases, the ex vivo data are an overestimation of the in vivo ones.

## 4. Discussion and Conclusions

Incomplete ablation is a significant concern in ablation therapy, as it can lead to tumor recurrence, particularly at the tumor margins [26,27,28]. Microwave ablation has shown a success rate of achieving complete ablation in 60–90% of tumors ranging from 3 to 5 cm in diameter [21,29,30]. However, there are indications that microwave ablation may be more susceptible to heat loss or heat-sink effects in vivo, compared to ex vivo animal studies and reference guides provided by ablation device manufacturers [19]. Previous research using an in vivo porcine model without tumor treatment demonstrated that the ablation zone reaches a plateau after a few minutes, likely due to thermal diffusion or negative heat flux caused by blood perfusion [31]. Heat diffusion increases with distance from the energy source, and underlying liver disease can affect this energy transfer.

In this study, it was observed that the dimensions of the ablation zone, specifically LAD, SAD, and AV, for an EMPRINT antenna were smaller in ex vivo tissue compared to those in in vivo tissue, up to a certain treatment energy. The observed trend reversal in the experimental data is also present in other antennas, such as AMICA and NeuWave Certus PR [18]. Regarding AMICA, the energy values at which the trend reversal takes place are 28, 1, 18 kJ for LAD, SAD and AV, respectively. Therefore, despite there being a similar trend here, the reversal occurred at different energy values. Particularly, the significantly different energy values at which the trend reversal occurred confirmed that the ablation pattern was different, with the AMICA antenna exhibiting a more oval-shaped ablation zone compared to that with the EMPRINT antenna. Regarding the Certus antenna, the trend reversal occurs at energy values of 20 kJ and 19 kJ for LAD and AV, respectively. However, no trend reversal is observed for SAD throughout the experimental range of energy levels (0–120 kJ). The trend reversal shown in the results and in the literature is due to the absence of blood during ex vivo experiments and its presence during in vivo experiments. In fact, blood absorbs a part of the energy transferred to the tissue during in vivo experiments, causing a slower transfer of heat into the tissue. The sphericity of the ablation zone is used as a measure to assess the uniformity of heating in all dimensions from the ablation antenna. The EMPRINT antenna is known for producing a more spherical ablation zone compared to that of other antennas from different manufacturers. The sphericity index of the EMPRINT antenna, calculated from the LAD and SAD, demonstrated high values with an average of 0.87 and 0.92 for ex vivo and in vivo, respectively. These values are consistent with the findings from other studies on the EMPRINT antenna [32,33]. However, the in vivo sphericity index calculated in this study was approximately 5.4% higher than the ex vivo-derived value. Statistical analysis confirmed that the presence of perfusion had an influence on the sphericity index values in in vivo tissue. The comparison of the EMPRINT, AMICA, and NeuWave Certus PR antennae revealed that the EMPRINT antenna induced the most spherical ablation zone. In fact, the mean sphericity index of the EMPRINT antenna was found to be, in vivo and ex vivo, 52.9% and 32.4% higher than that of AMICA, and 14.1% and 13.8% higher than that of Certus PR, respectively [18].

However, it is important to acknowledge the limitations of the present study. Firstly, the study was conducted retrospectively, and this introduced the possibility of confounding factors not being adequately accounted for. Additionally, the sample size of the study was relatively small, consisting of only 13 and 27 cases for in vivo and ex vivo data, respectively. Furthermore, combining power and time into a single independent variable of energy in the tissue may have resulted in a loss of data fidelity, since this can be intended as a pure qualitative parameter to be related with ablation zone sizes. It remains unclear whether time or power holds a disproportionate influence on determining the properties of ablation dimensions. To address these limitations, future research could consider conducting a prospective study with a larger sample size and carefully controlling both wattage and time variables. This would provide a more comprehensive understanding of the relative importance of these factors in the ablation process. In conclusion, based on the findings of this retrospective study involving 36 liver ablations using the AMICA antenna, it can be inferred that in vivo human liver ablation volumes were significantly smaller than the values obtained from ex vivo measurements when an energy level of at least 20 kJ was applied during the treatment.

## Figures and Tables

**Table 1:** Ablation volume for each energy range in ex vivo and in vivo experiments in the range of 0–24 kJ.

	Ex Vivo (*n* = 27)		In Vivo (*n* = 13)	
Energy (kJ)	Average (mm^3^)	Range (mm)	Average (mm^3^)	Range (mm)
0–11	3967	3026–5079	11,166	8836–13,210
12–18	7862	6842–9162	10,791	6925–14,137
19–24	13,786	10,618–16,964	12,332	7841–15,079

**Table 2:** Long ablated diameter (LAD) for each energy range in ex vivo and in vivo data in the range of 0–24 kJ.

	Ex Vivo (*n* = 27)		In Vivo (*n* = 13)	
Energy (kJ)	Average (mm^3^)	Range (mm)	Average (mm^3^)	Range (mm)
0–11	21.1	19.1–23.2	29.0	27.0–30.0
12–18	26.7	25.5–28.0	28.8	26.0–32.0
19–24	33.0	30.0–36.0	30.0	26.0–32.0

**Table 3:** Short ablated diameter (SAD) for each energy range in ex vivo and in vivo data in the range of 0–24 kJ.

	Ex Vivo (*n* = 27)		In Vivo (*n* = 13)	
Energy (kJ)	Average (mm^3^)	Range (mm)	Average (mm^3^)	Range (mm)
0–11	18.8	17.0–21.0	29.0	27.0–30.0
12–18	23.6	22.0–25.0	28.3	25.0–32.0
19–24	28.2	26.0–30.0	30.0	24.0–27.0

## Data Availability

The datasets analyzed during the current study are available from the corresponding author upon reasonable request.
